# Maternal effects and maternal selection arising from variation in allocation of free amino acid to eggs

**DOI:** 10.1002/ece3.1524

**Published:** 2015-05-29

**Authors:** Devi Newcombe, John Hunt, Christopher Mitchell, Allen J Moore

**Affiliations:** 1 Centre for Ecology and Conservation, University of ExeterCornwall Campus, Penryn, TR10 9EZ, UK; 2 Department of Genetics, University of GeorgiaAthens, Georgia, 30602

**Keywords:** Free amino acids, linear selection gradients, maternal effects, maternal selection, offspring performance, *Oncopeltus fasciatus*, performance surface, quadratic selection gradients

## Abstract

Maternal provisioning can have profound effects on offspring phenotypes, or maternal effects, especially early in life. One ubiquitous form of provisioning is in the makeup of egg. However, only a few studies examine the role of specific egg constituents in maternal effects, especially as they relate to maternal selection (a standardized selection gradient reflecting the covariance between maternal traits and offspring fitness). Here, we report on the evolutionary consequences of differences in maternal acquisition and allocation of amino acids to eggs. We manipulated acquisition by varying maternal diet (milkweed or sunflower) in the large milkweed bug, *Oncopeltus fasciatus*. Variation in allocation was detected by examining two source populations with different evolutionary histories and life-history response to sunflower as food. We measured amino acids composition in eggs in this 2 × 2 design and found significant effects of source population and maternal diet on egg and nymph mass and of source population, maternal diet, and their interaction on amino acid composition of eggs. We measured significant linear and quadratic maternal selection on offspring mass associated with variation in amino acid allocation. Visualizing the performance surface along the major axes of nonlinear selection and plotting the mean amino acid profile of eggs from each treatment onto the surface revealed a saddle-shaped fitness surface. While maternal selection appears to have influenced how females allocate amino acids, this maternal effect did not evolve equally in the two populations. Furthermore, none of the population means coincided with peak performance. Thus, we found that the composition of free amino acids in eggs was due to variation in both acquisition and allocation, which had significant fitness effects and created selection. However, although there can be an evolutionary response to novel food resources, females may be constrained from reaching phenotypic optima with regard to allocation of free amino acids.

## Introduction

Maternally provisioned egg constituents determine the initial resources available to the offspring for embryonic development so that maternal allocation of the quantity and quality of resources to the eggs can have profound effects on early offspring size and development, subsequent life history, and therefore evolution (Fox and Mousseau 1996; Mousseau and Fox 1998). However, the evolutionary consequences are not necessarily straightforward. Egg provisioning is a maternal environmental influence on offspring phenotype and thus a source of nutritionally based maternal effects (Rossiter 1991; Royle et al. 1999; Blount et al. 2002; Kyneb and Toft 2006). If mothers vary and this variation results in fitness differences among offspring, the provisioning of eggs also results in maternal selection (selection arising because offspring fitness is determined by the maternal phenotype; Kirkpatrick and Lande 1989). This suggests that understanding the sources and consequences of variation in egg constituents would help reveal mechanisms of maternal effects. However, studying egg quality can be difficult. Often, egg size is used as a proxy for egg quality and a source of the common environment for the embryo provided by the mother (Bernardo 1996; McIntyre and Gooding 2000). Although there are many studies investigating the relationship between maternal environment, egg size, and offspring performance traits (Mousseau and Dingle 1991; Mousseau and Fox 1998), few identify the specific aspects of egg traits that result in the maternal effect. Unfortunately, egg size is only a crude measure of maternal allocation of nutrient resources to offspring (McIntyre and Gooding 2000; Giron and Casas 2003; Lock et al. 2007) and may not be a reliable predictor for egg composition (Bernardo 1996; Giron and Casas 2003; Geister et al. 2008) or offspring fitness (McIntyre and Gooding 2000). Investigating effects of specific egg constituents is therefore important for understanding the full impact of maternal effects on evolution. Furthermore, measuring specific traits allowed us to determine both the cause of the maternal effect and maternal selection, which arises whenever variation in the fitness of the offspring is influenced by variation in the maternal traits (Kirkpatrick and Lande 1989).

The most commonly studied maternally allocated egg constituents include hormones, steroids, antibodies, carotenoids, and vitamins, all of which are studied especially well in birds and all have been shown to affect offspring phenotypes (Blount et al. 2002; Verboven et al. 2003; McGraw et al. 2005; Uller and Olsson 2006; Uller et al. 2007; Groothuis and Schwabl 2008; Müller et al. 2012; Postma et al. 2014). A relatively poorly studied but fundamental and ubiquitous constituent allocated by mothers to the eggs is amino acids. This omission is striking because amino acids are the building blocks of proteins and are therefore vital to the growth and development of offspring. For example, amino acids play an important role in the reproductive biology of herbivorous insects and having the right balance of amino acids may be required to promote embryogenesis (Uchida 1993). Female butterflies (Alm et al. 1990; Mevi-Schütz and Erhardt 2003, 2005; Cahenzli and Erhardt 2012) and honey bees (Alm et al. 1990) prefer nectars with amino acids added artificially when raised on a low-quality larval diet, which may be a compensatory mechanism for overcoming nutritive deficiencies in the juvenile stage. In Lepidoptera, amino acids from both larval and maternal diets are incorporated into eggs (O'Brien et al. 2002, 2003, 2005) and amino acids enhance female fecundity (Mevi-Schütz and Erhardt 2005; Cahenzli and Erhardt 2012). Around 10 amino acids are considered to be essential for the growth and development of insects (Sandström and Moran 1999), but the specific amino acids and their concentrations required vary within and between species (House 1962), suggesting patterns of allocation have evolved. Yet, while many studies have investigated the relationship between dietary amino acids and adult female fecundity, few studies have explored the relationship between adult female amino acid allocation and offspring fitness (Geister et al. 2008).

The aim of this study was to investigate whether maternal effects and maternal selection arise from variation in the allocation of free amino acids to eggs. Given the role of amino acids in embryonic development, we predicted that amino acids would be significant maternal effects and create significant maternal selection. Our previous work on the milkweed bug, *Oncopeltus fasciatus*, has shown that female diet affects offspring fitness through maternal effects (Newcombe et al. 2015). In addition, maternal effects are known to be of importance in *O. fasciatus* life history (Groeters and Dingle 1987, 1988). Here, we test the hypothesis that there will be variation in free amino acid concentrations in the egg arising from differences in acquisition and allocation associated with different diets, that these differences will lead to maternal effects, and that maternal selection will arise because offspring fitness is affected by maternal acquisition and allocation of amino acids. To test our hypothesis, we studied two diets (milkweed and sunflower) and two source populations. We predicted that these maternal effects would evolve in accordance with selection arising from variation in acquisition of the different diets. Thus, if maternal effects are adaptive, we predicted that a source population that had evolved to use sunflower would be nearer a fitness maximum for offspring under a sunflower diet than a source population that had never experienced sunflower. The source population adapted to milkweed only should be nearer the fitness maximum for milkweed.

We experimentally manipulated maternal diet in two different source populations of the large milkweed bug, *Oncopeltus fasciatus*, and examined how these factors influenced amino acid allocation to eggs. We further examined how the amino acid profiles of the eggs affected offspring performance. The two source populations we studied have different evolutionary histories and are adapted to different diets (Newcombe et al. 2015). One (Kentucky) is formed of individuals collected from the wild. This species and therefore these individuals are highly specialized to use milkweed seeds, *Asclepias syriaca* (Apocynaceae), as its diet (Beck et al. 1958; Sauer and Feir 1973; Feir 1974). The other source population (Laboratory) was derived from this wild population some time ago but has been exclusively reared on sunflower seeds, *Helianthus annuus*, for over 400 generations in the laboratory and has an expanded diet (Attisano et al.^2012^; Newcombe et al. 2015). Milkweed and sunflower differ considerably in composition and nutrition (Robinson 1978; Hojiila-Evangelista et al. 2009), and this is reflected in differences in the fatty acid composition of body lipids when adults are reared on each diet (Nation and Bowers 1982).

We first examined free amino acid content of whole eggs to determine whether changes to maternal diet influence quantitative differences in amino acid profiles of eggs. We next asked whether the patterns we observed depended on the population examined, as well as an interaction between maternal diet and population, to examine the potential for allocation to evolve. We then estimated the strength and form of linear (directional selection) and nonlinear maternal selection (Lande and Arnold 1983; Gershman et al. 2012) and used canonical analysis to find the major axes of the quadratic response surface (Blows et al. 2003) to determine how amino acid allocation influenced offspring performance. Finally, we used thin-plate splines to visualize the performance surface along the major axes of nonlinear selection and plot the mean amino acid profile of eggs from each treatment onto the performance (fitness) surface to determine the proximity of these treatment means to peak performance (Blows et al. 2003; Brooks et al. 2005).

Our study design capitalized on the availability of an experimental source population of *O. fasciatus* that has evolved to use a novel host food that we can compare to the “natural” source population (individuals collected from the wild and still maintained on its natural food source). We hypothesized that evolving to use a new food will also involve evolution of maternal effects because the nutritional composition of plants can differ. In nature, the large milkweed bug, *O. fasciatus*, is an herbivorous insect that is highly specialized to feed on milkweed seeds from the genus *Asclepias*. Milkweed and diet play an important role in the life history and reproductive biology of *O. fasciatus* (Beck et al. 1958; Gordon and Gordon 1971; Ralph 1976; Isman 1977; Chaplin 1980; Slansky 1980a,b; Blakley 1981). As maternal diet can have significant consequences for the provisioning of egg constituents (Grindstaff et al. 2003; Müller et al. 2012), we predicted that when challenged with a novel food source a specialist insect such as *O. fasciatus* will alter allocation of resources into the oocytes. To the extent that early nutrition influences offspring, this differential allocation of resources to the eggs should have an effect on the developing embryo. However, because only two exist, we treat source population as a fixed factor, and all of our inferences are limited to these specific sources and are not necessarily generalizable to other populations (if they were to exist).

## Materials and Methods

### Study system

The large milkweed bug, *O. fasciatus*, is found across North America and in parts of central and northern South America (Feir 1974). *Oncopeltus fasciatus* feed and reproduce mainly on milkweed plants from the family Asclepiadaceae, which contain toxic cardiac glycosides (cardenolides) (Feir 1974; Ralph 1976). Although milkweed is the preferred and natural host, in the laboratory, *O. fasciatus* can be reared on a variety of food sources that differ greatly in composition, including sunflower, cashew, pumpkin seeds, and peanuts (Beck et al. 1958; Gordon and Gordon 1971; Feir 1974; Scudder et al. 1986). While initial performance is poor on these hosts, improved performance evolves within 10 generations (Gordon and Gordon 1971; Feir 1974).

To test for differences in maternal allocation, we used two source populations of *O. fasciatus* that have been reared and maintained on different host diets. Note that we have only two sources (there are only two) and so these are analyzed as fixed factors. Replication occurs at the level of individuals within these populations. Because they are fixed factors, while we can detect significant effects we cannot attribute these to general influences; any differences are associated with all differences between the source populations. Thus, while we interpret differences between sources as arising from evolution, we can only infer this and cannot determine whether the differences are due to drift or adaptive evolution. Nonetheless, given the large differences in diet and their effects, we can have some confidence that much if not most of the differences we see are due to diet (Newcombe et al. 2013, 2015). Importantly, however, not all dietary differences are reflected in the offspring. The two populations do not differ in allocation of cardenolides from milkweed even though the Laboratory population have not seen milkweed for over 400 generations (Newcombe et al. 2013).

One source population derived from individuals was collected from the wild at the University of Kentucky Arboretum, Lexington, KY, USA. In nature, milkweed plants from the genus Asclepias (Apocynaceae) are the preferred and natural host for *O. fasciatus*. We maintained this source population on a diet of dried milkweed seeds, *Asclepias syriaca*, purchased from Educational Science, League City, TX, USA. The other source population was supplied from Carolina Biological Supply House (Burlington, NC). This is a long-standing Laboratory population, which has been reared on dehusked sunflower seeds, *Helianthus annuus*, (purchased from Goodness Direct) for over 400 generations (sunflower population). Both source populations were reared under common garden laboratory conditions in very large mass colonies in multiple boxes. The Kentucky (milkweed) colonies were maintained for at least three generations in the laboratory before used in any experiments, to minimize carry-over maternal effects from the field. Colonies are kept in incubators at 25°C with a light: dark regime of 16:8. We used upturned glass jars (with a base made from paper towels and the base of a petri dish) filled with demineralized water as water receptacles and provided fresh seeds (as appropriate), water, and cotton wool (oviposition substrate) as necessary.

### Experimental populations and rearing

We created multiple colonies of each source population and reared them in 28 × 16 × 9 cm boxes so that all individuals were derived from the same generation. Each colony was reared in very large numbers and maintained on either milkweed seeds or sunflower seeds, thereby creating four treatments: (1) Kentucky-derived individuals on milkweed seeds (KYMW); (2) Kentucky-derived individuals on sunflower seeds (KYSF); (3) Laboratory-derived individuals on milkweed seeds (LABMW); and (4) Laboratory-derived individuals on sunflower seeds (LABSF). These colonies were housed in a single incubator at 25°C with a light: dark regime 18:6 h. Boxes were routinely moved around the incubator.

### Experimental design

As it is difficult to sex nymphs until late instar stages, newly eclosed (virgin) adults were collected daily from each of the source populations. These individuals were then removed to individual containers. Females and males were housed as per their treatment and collection date in either standard petri dishes or small boxes (11 × 11 × 3 cm). We only retained males if they had been raised on their original host diet, that is, Kentucky males raised on milkweed and Laboratory males raised on sunflower. Adults were provided with their allocated diet (sunflower or milkweed seeds) ad libitum, and a cotton wick moistened with demineralized water. Seeds and water were replenished as necessary.

To ensure that adults were fully sexually mature, we mated females when they were between 7 and 10 days old and males were between 5 and 10 days old (see Gordon and Loher 1968). We only mated females, regardless of diet treatment, with a male from her respective source population that had been reared on their original source population diet. Each female was only mated with one male, and to encourage egg production and ensure fertilization, we left pairs together for 72 h. Mated pairs were housed in standard petri dishes and maintained on the allocated diet of the female. Moist cotton wicks were also provided and changed as required. Males were discarded after mating.

Upon separation of the sexes, we counted the numbers of eggs laid by each female and up to 10 eggs were selected for weighing and amino acid analysis. The rest of the eggs from the clutch were kept for hatching. We used a Mettler Toledo UMX2 microbalance to weigh the eggs and nymphs. A small piece of foil was used as a weigh boat. Weighed eggs were frozen at −80°C until analysis could be conducted. Clutches that were found to be infertile (see below) were removed from any analysis. We collected eggs for amino acid analysis from a total of 201 females (33 from KYMW, 48 from KYSF, 50 from LABMW, and 69 from LABSF).

We placed eggs kept for hatching in cotton wool in an incubator with L:D 18:6 at 25°C (±1°). We checked daily from day 5 for hatching and recorded the date of hatching and the number of hatchlings. Nymphs were chilled in a refrigerator for up to one hour before weighing. If eggs did not hatch by day 8, then we scored the eggs as unfertilized, and the corresponding eggs from that clutch that had been set aside for amino acid analysis were not included in any further analysis.

### Amino acid extraction

We prepared eggs for analysis following modified methods of Gelman et al. (2000). We collected 10 eggs from each female and placed them in an Eppendorf tube and pipetted 100 *μ*L of 75% ethanol into each sample. We then sonicated samples for 3 min, then mashed with a manual pestle, and washed again with 100 *μ*L of 75% ethanol. We then pulse-vortexed samples for 5 sec and then placed them on ice for 30 min. Following this, we centrifuged samples for 10 min at 4°C at maximum speed. We then placed samples in liquid nitrogen, removed the supernatant, and placed this into fresh Eppendorf tubes. We stored samples at −80°C until the amino acid analysis.

To analyze amino acids, we used a Phenomenex (Torrance, CA, USA) EZ:faast™ kit for free amino acid analysis. We pipetted 100 *μ*L of each sample into a sample preparation vial along with 100 *μ*L of Reagent 1 (internal standard solution; norvaline 0.2 nmol/L, N-propanol 10%). The solution was then passed slowly through a sorbent tip attached to a 1.5-mL syringe. Any liquid passed into the barrel and not kept within the sorbent tip was discarded. We next pipetted 100 *μ*L of Reagent 2 (wash solution; N-propanol) into the sample preparation vial, which was then drawn slowly through the sorbent tip and into the syringe barrel. Liquid that accumulated in the barrel was again discarded, leaving only the solution contained within the tip. Next, we pipetted 200 *μ*L of freshly prepared eluting medium (a 3:2 mix of sodium hydroxide and N-propanol) into the preparation vial. After drawing air into the barrel of a 0.6-mL syringe, the syringe was attached to the sorbent tip and the eluting medium was slowly passed through until the liquid reached the filter plug within the sorbent tip, thereby wetting the sorbent with the eluting medium. The liquid and sorbent particles were then ejected out of the tip and into the sample preparation vial until only the filter disk remained within the empty tip. Using the adjustable Drummond Dialamatic microdispenser, we transferred 50 *μ*L of Reagent 4 (chloroform) into preparation vial. We then vortexed the vial for 5–8 sec in pulse mode at 80% of maximum speed. The reaction was then left to proceed for at least 1 min. The vials were then vortexed again for 5 sec and left for the reaction to proceed for another minute. We then used the microdispenser to transfer 100 *μ*L of Reagent 5 (iso-octane), vortexing the vial for 5 sec, and leaving the reaction to stand for 1 min. This allowed separate layers to develop, and 50–100 *μ*L of the organic layer was pipetted into the insert of an autosampler vial. We then slowly evaporated the solvent under a nitrogen stream. Finally, we redissolved the amino acid derivatives in 100 *μ*L of Reagent 6 (hydrochloric acid) and vortexed for 10 sec. Vials were capped and stored at −80°C until analysis using the GC/MS.

### Quantification of free amino acid composition

We measured the following 17 amino acids using EZ:faast™: alanine, glycine, leucine, isoleucine, threonine, serine, proline, asparagine, aspartic acid, methionine, glutamic acid, phenylalanine, glutamine, lysine, tyrosine, and tryptophan. The amino acids *α*-aminobutyric acid, valine, cystathionine, orthinine, and glycyl-proline were not analyzed, as they were not detected in all of our samples. Likewise, sarcosine, *β*-aminoisobutyric acid, alloleucine, thiaproline, 4-hydroxyproline, hydroxylysine, and proline-hydroxyproline were not detected in any of our samples and are not considered further.

### Statistical analysis

We conducted all statistical analyses using IBM SPSS Statistics version 19 (IBM Corporation, Armonk, NY, USA). We divided the amino acid values by the total mass of eggs from each sample to standardize amino acid values with clutch size from each female. For each female, we used mean egg mass and mean offspring (nymph hatchling) mass in our statistical analyses.

Given the number of amino acids measured, we used principal component analysis (PCA) to reduce the number of individual variables into a tractable number of dimensions (Tabachnick and Fidell 1989; Gershman et al. 2012). We extracted the principal components (PCs) using the correlation (rather than covariance) matrix, to minimize the effects of differences in scale on the PCs extracted (Tabachnick and Fidell 1989). PCs with an eigenvalue of greater than 1 were retained for further analysis (Gershman et al. 2012). We interpret factor loadings of 0.45 or above as biologically relevant. We removed four multivariate outliers prior to analysis, based on their Mahalanobis distances (Tabachnick and Fidell 1989). Once the PCs were extracted, we used a multivariate analysis of covariance to test for any effects of source population and maternal diet, as well as their interaction on the PCs describing variation in amino acids, including female body size as a covariate. We used univariate analysis of covariance to determine which PCs contributed to any overall significant multivariate effects.

### Maternal selection and performance surface estimation

Maternal selection occurs when the fitness of the offspring is at least partially determined by maternal traits (Kirkpatrick and Lande 1989). We therefore used a conventional multivariate selection analysis based on multiple regression analysis (Lande and Arnold 1983; Gershman et al. 2012) to investigate maternal selection arising from amino acid profiles of eggs influencing offspring performance. The performance traits we measured (egg mass and hatchling mass) relate directly to offspring fitness (viability; Newcombe et al. 2015) as recommended by Lande and Arnold (1983). All PC scores were standardized to a mean of zero and standard deviation of 1 (Lande and Arnold 1983). We then fitted a linear regression including the PCs describing the amino acid composition of eggs and offspring performance to estimate the vector of standardized linear selection gradients (***β***) for each relative performance measure. A quadratic regression model including all the linear, quadratic, and cross-product terms was then used to estimate the matrix of nonlinear selection gradients (***γ***). Quadratic regression coefficients were doubled (Stinchcombe et al. 2008).

The strength of nonlinear selection is greatly underestimated if the size and significance of ***γ*** terms are interpreted individually (Blows et al. 2003). We therefore used canonical analysis (Phillips and Arnold 1989) to locate the major axes of nonlinear selection acting along the performance surface for each offspring measurement. The strength of linear selection along each of the eigenvectors (***m***_***i***_) is given by theta (***θ***_***i***_), and the strength of nonlinear selection is given by their eigenvalues (***λ***_***i***_). We estimated ***θ***_***i***_ and ***λ***_***i***_ using the double regression method of Bisgaard and Ankenman (1996).

As our offspring performance measures were not normally distributed, we tested the significance of our standardized selection gradients using a resampling procedure where relative offspring performance measures were shuffled randomly across individuals in the dataset to obtain a null distribution for each selection gradient where there is no relationship between the PCs describing amino acids in the eggs and offspring performance. Probabilities are the number of times (of 9999 permutations) in which the gradient pseudo-estimate was equal to or less than the original estimated gradient. We conducted separate randomization tests for the multiple regression models for linear selection and for the full quadratic model. We used the same resampling procedure to assess the significance of ***θ***_***i***_ and ***λ***_***i***_ for each eigenvector after the canonical analysis of ***γ***.

We used thin-plate splines (Green and Silverman 1994) to visualize the major axes of the performance surface extracted from the canonical analysis of ***γ***. We used the *Tps* function in the FIELDS function of R (v. 2.12.2, http://www.r-project.org) to fit the thin-plate splines and to visualize them in contour view. We used the value of the smoothing parameter (***λ** *= 0.021) that minimized the generalized cross-validation scores when fitting the thin-plate splines. We used a sequential model building approach (Draper and John 1988; Chenoweth and Blows 2005) to determine whether maternal selection on the amino acid composition of eggs differed across our four treatments.

## Results

### Offspring performance

**Figure 1:**
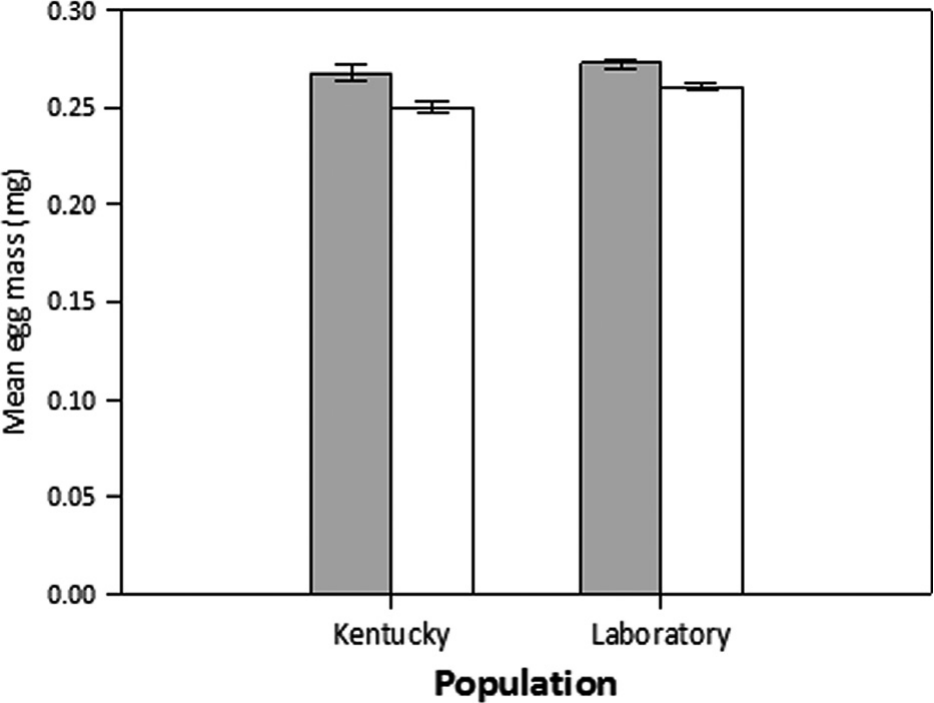
Differences in egg mass arising from population and diet. Mean egg mass (±SE) across individuals from the Kentucky and Laboratory source populations when females reproduce on milkweed (gray bars) and sunflower (white bars) diets.

Source population (*F*_1,193_ = 10.450, *P *=* *0.0014) and maternal diet (*F*_1,193_ = 33.577, *P *<* *0.0001) had a significant effect on egg mass, with a marginally significant interaction between source population and maternal diet (*F*_1,193_ = 3.727, *P *=* *0.055). The Laboratory-derived individuals laid heavier eggs than the Kentucky-derived individuals (Fig.1). All females fed milkweed seeds laid heavier eggs than females fed sunflower seeds (Fig.1). Female size significantly covaried with egg mass (*F*_1,192_ = 9.989, *P *=* *0.0018), as bigger females had heavier eggs.

**Figure 2:**
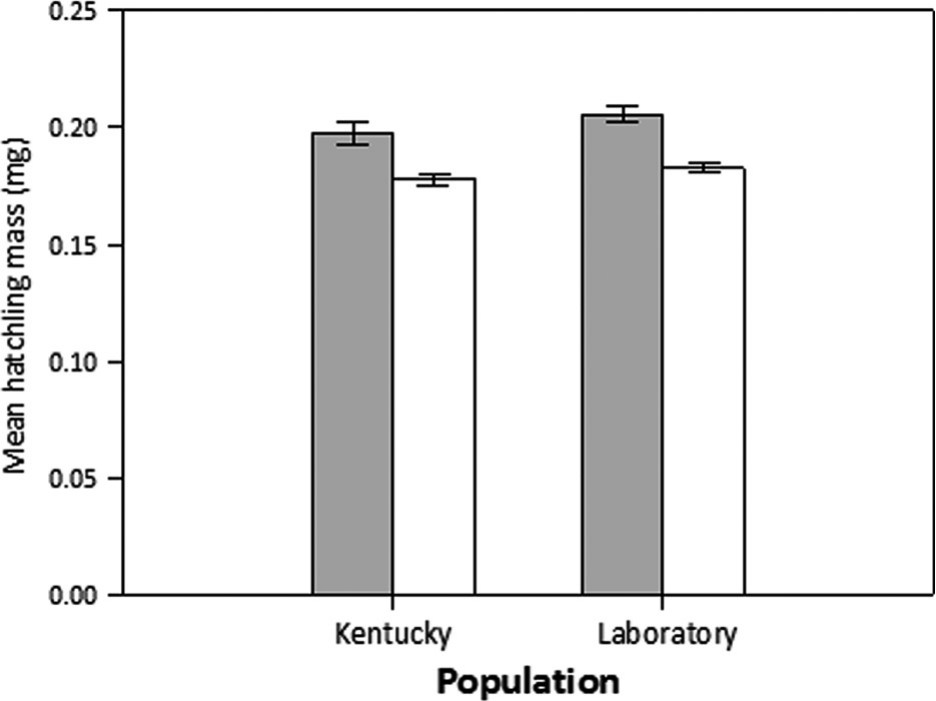
Differences in hatchling mass arising from population and diet. Mean hatchling mass (±SE) across individuals from the Kentucky and Laboratory source populations when females reproduce on milkweed (gray bars) and sunflower (white bars) diets.

There was a statistically significant effect of source population on hatchling mass (*F*_1,191_ = 4.607, *P *=* *0.033). Maternal diet was again a highly significant effect (*F*_1,191_ = 40.4601, *P *=* *0.0001). There was no significant interaction between these main effects (*F*_1,191_ = 0.0137, *P *=* *0.907). Consistent with the effects on egg mass, hatchlings from females fed milkweed were significantly larger than hatchlings born to females fed sunflower (Fig.2). We found no significant covariance between female size and hatchling mass (*F*_1,191_ = 1.607, *P *=* *0.207).

### Maternal effects – free amino acid composition of eggs

Our analysis of free amino acid content of the eggs identified variation in the composition of 16 amino acids. We found four PCs with eigenvalues >1 that collectively explained 66% of the total variation in free amino acid content of our egg samples (Table1). PC1 explains 29.85% of the variance of amino acid content and is positively loaded with most of the amino acids present (except proline) and significantly related to amounts of leucine, isoleucine, threonine, serine, asparagine, methionine, glutamic acid, phenylalanine, glutamine, and lysine, but not alanine, glycine, aspartic acid, tyrosine, and tryptophan. This suggests that these amino acids constitute the free amino acid pool of the eggs at this stage of development, and PC1 reflects overall quantitative differences in the total amount of free amino acids. PC2 explains 16.42% of the variance in amino acid composition in the eggs and demonstrates an opposite relationship between glycine, proline, tyrosine and tryptophan (positive loadings) and glutamic acid (negative loadings). PC2 therefore describes the amounts of those not included in PC1 and is therefore complementary. PC3 accounts for a further 11.73% of the variation with opposite relationships between alanine, aspartic acid and tryptophan (positive loadings) and threonine and asparagine (negative loadings). PC4 accounts for 7.9% of variation in amino acid composition with opposite relationships between asparagine and glutamine (positive loadings) and serine (negative loading). Thus, PC3 and PC4 indicate differences in the specific composition of free amino acids and reflect variation in allocation patterns, independent of quantitative differences.

**Table 1:** Principal component analysis (PCA) of the free amino acid composition of eggs in *Oncopeltus fasciatus*. We retained PCs with eigenvalues exceeding 1 for further analysis and interpret factor loadings of ¦0.45¦ or above as biologically important (in bold). Amino acids in italics are essential amino acids

	Principal component			
	PC1	PC2	PC3	PC4
Eigenvalues	4.775	2.663	1.877	1.268
% Variance explained	29.85	16.46	11.73	7.93
Amino acids				
Alanine^1^	0.110	0.258	**0.522**	0.311
Glycine	0.098	**0.844**	−0.181	0.024
*Leucine*	**0.755**	0.194	0.206	−0.102
*Isoleucine*^1^	**0.843**	0.293	−0.178	−0.093
*Threonine*^1^	**0.652**	0.105	−**0.575**	0.139
Serine^1^	**0.476**	−0.108	0.158	−**0.616**
Proline	−0.122	**0.826**	0.057	−0.037
Asparagine	**0.509**	0.113	−**0.478**	**0.480**
Aspartic acid	0.178	−0.158	**0.677**	0.307
*Methionine*^1^	**0.654**	−0.070	−0.213	-0.346
Glutamic acid	**0.764**	−**0.412**	0.140	0.052
*Phenylalanine*	**0.694**	0.110	0.311	0.208
Glutamine	**0.619**	−0.187	−0.083	**0.423**
*Lysine*^1^	**0.702**	−0.339	0.308	−0.154
Tyrosine^2^	0.377	0.393	0.032	−0.242
*Tryptophan*	0.057	**0.705**	**0.434**	−0.018

Those amino acids marked with

1 are potentially important intermediaries for the mevalonate cycle and those marked with

2 are biosynthesized from phenylalanine.

### Acquisition and allocation of amino acid composition of eggs

**Figure 3:**
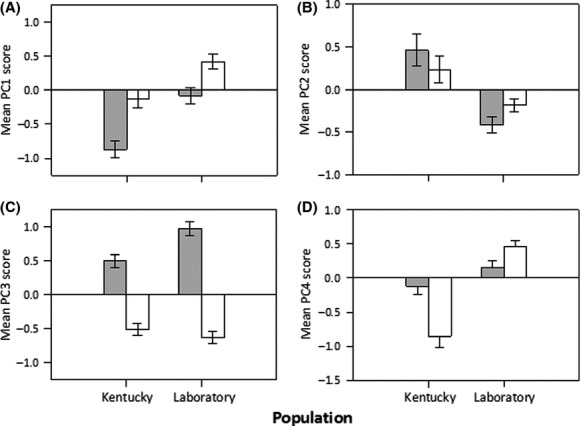
Differences in free amino acid composition of eggs arising from population and diet. Mean PC scores (±SE) describing the amino acid composition of eggs across individuals from the Kentucky and Laboratory source populations when females reproduce after feeding on milkweed (gray bars) or sunflower (white bars) diets. (A) PC1, (B) PC2, (C) PC3, and (D) PC4.

Overall, there was a significant effect of source population and maternal diet, as well as a significant interaction between these main effects, for all four PCs describing the amino acid composition of the eggs (Table2). In contrast, the amino acid composition of eggs did not significantly vary with female size (Table2). Given the overall significance of our MANCOVA, we investigated the univariate effects on each PC using ANCOVA. The overall multivariate effect of source population was driven by significant differences in PC1, PC2, and PC4 (Table2; Fig.3). On average, PC1 and PC4 values were higher for individuals derived from the Laboratory source population, but this pattern was reversed for PC2 (Fig.3). The overall multivariate effect of maternal diet was driven by significant differences in PC1 and PC3 (Table2; Fig.3). On average, PC1 values were higher on the sunflower diet than the milkweed diet, while the reverse pattern was observed for PC3 (Fig.3). The overall multivariate effect of the interaction between source population and maternal diet was driven by significant differences in PC2, PC3, and PC4 (Table2; Fig.3). The significant interaction for PC2 occurs because there was a difference in PC2 values across maternal diets in individuals from the Laboratory source population but not the Kentucky source population (Fig.3). The significant interaction for PC3 occurs because although PC3 values are higher for milkweed than the sunflower diet in both, this difference is more pronounced in individuals from the Laboratory population (Fig.3). The significant interaction for PC4 occurs because of the opposing effects maternal diet have on PC4 values across source populations (Fig.3).

**Table 2:** Multivariate analysis of variance (MANOVA) examining the effects of source population (effect A: Kentucky or Laboratory) and maternal diet (effect B: milkweed or sunflower), as well as the interaction between these main effects, on four PCs describing free amino acid composition of the eggs of *Oncopeltus fasciatus*. We follow the overall MANOVA with univariate analysis of covariance (ANCOVA) to determine how each of the PCs contributes to the overall multivariate effects

Model term	MANOVA		
	Pillai's Trace	*F*_4,188_	*P* value
Source population (A)	0.426	34.870	0.000
Maternal diet (B)	0.553	58.063	0.000
A × B	0.120	6.379	0.000
Female size	0.029	1.415	0.230

	Univariate ANCOVAs		
	*F*	df	*P*
Source population (A)			
PC1	31.666	1,191	0.0001
PC2	25.933	1,191	0.0001
PC3	3.630	1,191	0.058
PC4	42.826	1,191	0.0001
Maternal diet (B)			
PC1	21.639	1,191	0.0001
PC2	0.114	1,191	0.736
PC3	171.989	1,191	0.0001
PC4	2.005	1,191	0.158
A × B			
PC1	0.430	1,191	0.513
PC2	4.664	1,191	0.032
PC3	7.440	1,191	0.007
PC4	16.161	1,191	0.0001
Female size			
PC1	1.963	1,191	0.163
PC2	2.366	1,191	0.126
PC3	1.143	1,191	0.286
PC4	0.845	1,191	0.359

### Maternal selection and performance surface

**Figure 4:**
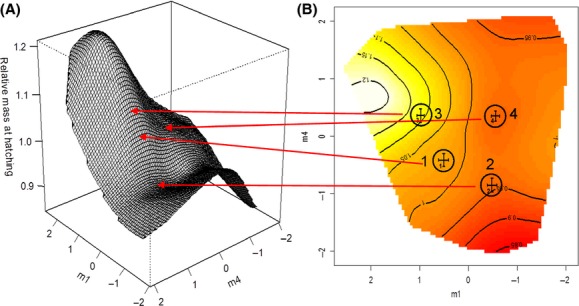
Performance surfaces for two populations of *Oncopeltus fasciatus* raised on two different diets. Thin-plate spline (A) perspective and (B) contour view visualization of the performance surface along the two major axes of nonlinear selection (m_1_ and m_4_). m_1_ and m_4_ represent the combinations of principal components (PCs) that explain the greatest nonlinear variation for hatchling mass. The combination of a positive eigenvalue on m_1_ (disruptive selection) and negative eigenvalue on m_4_ (stabilizing selection) (see Table4) indicates the presence of a multivariate saddle on the response surface. Mean m scores of each treatment are plotted onto the performance surface (4B). Performance peaks are found at high values of m_1_ and intermediate values of m_4_. In (B) the mean m_1_ and m_4_ scores for each treatment combination are provided where 1 = KYMW; 2 = KYSF; 3 = LABMW; and 4 = LABSF. Individuals from the Laboratory source population reared on milkweed (3) were closest to the performance peak followed by the individuals from the Kentucky source population reared on milkweed (1), individuals from the Laboratory source population reared on sunflower (4) with the Kentucky population reared on sunflower (2) found at the lowest point.

None of the standardized selection gradients for the relationship between the PCs describing amino acid composition of the eggs (the maternal trait) and hatching success (the correlate of offspring fitness) were significant (Table3) nor were the eigenvectors extracted from the canonical analysis of *γ* significant (Table4). In contrast, we found that there was significant positive linear selection for PC3 and PC4, as well as significant quadratic (stabilizing) selection on PC3 (Table4) associated with offspring mass (an alternative correlate of offspring fitness). There was also significant correlational selection for negative covariance between PC1 and PC3, and between PC2 and PC3 (Table4). Canonical analysis of the *λ* matrix found three eigenvectors with significant linear selection on **m**_**1**_ (heavily weighted by PC3 with decreased values of PC1 and PC2), **m**_**2**_ (heavily weighted by PC2 with decreased values of PC1), and **m**_**4**_ (heavily weighted by PC4) (Table4). Two eigenvectors, **m**_**1**_ and **m**_**4**_, were found to have significant quadratic selection acting along them (Table4). The positive eigenvalue of **m**_**1**_ indicates disruptive selection operating along this eigenvector, and the negative eigenvalue for **m**_**4**_ indicates stabilizing selection. This performance surface can be visualized as a thin-plate spline in Fig.4. The combination of positive and negative eigenvalues for these eigenvectors indicates the presence of a multivariate saddle on the response surface with a pronounced peak of performance at high positive values of **m**_**1**_ and intermediate values of **m**_**4**_ (Fig.4). We plotted the mean **m** scores for each treatment on the contour view of the performance surface (Fig.4B) to determine the proximity of our treatments to the performance peak. The Laboratory source population reared on milkweed was the closest to the performance peak, followed by the Kentucky source population reared on milkweed, the Laboratory source population reared on sunflower, and then the Kentucky source population reared on sunflower (Fig.4B).

**Table 3:** The vector of standardized linear selection gradients (*β*) and the matrix of standardized quadratic and correlational selection gradients (*γ*) for the four PCs describing the amino acid composition of the eggs in *Oncopeltus fasciatus* and their effects on (A) offspring hatching success and (B) hatchling mass

	*β*	*γ*			
		PC1	PC2	PC3	PC4
(A)					
PC1	−0.004	0.002			
PC2	−0.007	−0.045	0.026		
PC3	−0.004	−0.011	0.030	0.018	
PC4	−0.015	−0.026	0.009	−0.038	−0.028
(B)					
PC1	−0.015	−0.010			
PC2	−0.006	0.003	0.008		
PC3	0.051***	−0.028*	−0.023*	0.016	
PC4	0.029**	−0.015	0.003	−0.003	−0.030*

Randomization test:

* *P *<* *0.05,

** *P *<* *0.01,

*** *P *<* *0.001.

**Table 4:** The M matrix of eigenvectors from the canonical analysis of *γ* for the four PCs describing the amino acid composition of the eggs in *Oncopeltus fasciatus* and their effects on (A) offspring hatching success and (B) hatchling mass. The linear (*θ*_*i*_) and quadratic (*λ*_*i*_) gradients of selection along each eigenvector are given in the last two columns. The quadratic selection gradient (*λ*_*i*_) of each eigenvector (m_*i*_) is equivalent to the eigenvalue

	M				Selection	
	PC1	PC2	PC3	PC4	*θ*_*i*_	*λ*_*i*_
(A)						
**m**_**1**_	−0.515	0.728	0.452	0.027	−0.005	0.038
**m**_**2**_	0.387	−0.140	0.702	−0.581	0.005	0.018
**m**_**3**_	0.667	0.669	−0.314	−0.096	−0.005	−0.017
**m**_**4**_	0.375	−0.045	0.453	0.808	−0.015	−0.031
(B)						
**m**_**1**_	−0.417	−0.492	0.763	0.033	0.049***	0.023**
**m**_**2**_	−0.567	0.743	0.155	0.319	0.021*	0.001
**m**_**3**_	0.379	0.447	0.522	−0.620	0.001	−0.010
**m**_**4**_	0.600	0.078	0.348	0.716	0.029**	−0.024**

Randomization test:

* *P *<* *0.05,

** *P *<* *0.01,

*** *P *<* *0.001.

## Discussion

In this study, both diet and source population influenced egg size and hatchling mass, and while there is overlap in these effects, there are also differences. To understand whether these differences reflected allocation or acquisition, we specifically examined how the free amino acid content of eggs varied in response to differences associated with different source populations and different maternal diets, and the importance of these differences by analyzing maternal selection and performance surfaces associated with variation in amino acid composition. We found that the amino acid profiles of eggs differed between the populations, independent of food type, suggesting evolved differences in patterns of allocation. We found that the maternal diet, or differences in acquisition, also influenced these profiles. There was also a significant interaction between population and diet, suggesting that the source populations differ in how they allocate amino acids when challenged with different food sources; that is, the two populations have evolved different maternal effects. These results may be expected as these two populations have persisted on different diets (i.e., either milkweed seeds or sunflower seeds) for hundreds of generations. Our analysis of maternal selection and visual inspection of the two populations on the performance (fitness) surface (using hatchling mass as a proxy for fitness) suggests that the Laboratory population is better adapted to sunflower seeds, consistent with its evolutionary history, but that both populations are still best adapted to milkweed as a food source.

### Maternal acquisition and allocation

If acquisition were the most important determinant of maternal effects, given the different composition of the seeds (Robinson 1978; Hojiila-Evangelista et al. 2009), we predicted food type would be the major influence on amino acid composition of eggs. We found that, as expected, the overall quantity of amino acids (PC1 and PC2) reflected the maternal diet. In addition, PC3 and PC4, both of which suggest differences in the specific composition of free amino acids, were influenced by diet. Other studies have also shown, or implied that variation in maternal host diet may lead to variation in egg composition and perhaps maternal allocation. For example, Rossiter et al. (1993) demonstrated significant maternal effect evolution in response to diet in the gypsy moth, *Lymantria dispar*. They found that temporal and spatial variation in parental nutritional experience can lead to maternal variation in allocation of vitellogenin into eggs, resulting in variation in gene expression and phenotypic plasticity. In the eggs of the seed beetle, *Stator limbatus*, differences in egg color between females fed different host diets suggest differential allocation of resources through maternal host diet effects (Fox et al. 1995). They also found that maternal host effects in offspring performance traits. These results are perhaps unsurprising, as the resources needed to allocate to eggs are likely to be acquired through the maternal diet. Thus, the importance of host plant on maternal effects is likely to be considerable.

Of more evolutionary importance are patterns of allocation, which underlie adaptive maternal effects. Acquisition and allocation are often difficult to separate, but our experimental design allows us to tease these apart because we manipulated the food available to two different source populations, which themselves differ in their ability to utilize different food sources (Newcombe et al. 2015). We predicted that if allocation differences evolved, adaptively or not, source populations would differ regardless of the food type. In our study, the differences we see in PC1, PC3, and PC4 in the different source populations suggest that evolutionary history influence patterns of allocation given both sources had access to the same food. Thus, patterns of allocation were not simply a function of available resources from the food. This suggests that as populations evolve in response to different host use, maternal effects evolve alongside this dietary shift. We suggest this will occur because of maternal selection (below). This is further supported by the differences in allocation of amino acids, resulting in differential utilization of egg amino acid profiles reflected in the significant source population by diet interaction term. There was a significant interaction for all of the composition characters, PC2, PC3, and PC4, indicating that even given the same resources populations with different evolutionary histories differentially allocate amino acids into their eggs. Our results also support the suggestion of O'Brien et al. (2002, 2005) that understanding nutrient allocation should illuminate life-history tradeoffs, and investigating amino acid nutrition should help clarify the evolution of life histories and dietary specialization.

### Maternal selection

Given the differences in allocation we see among individuals derived from populations with different evolutionary histories, we also wished to know whether these allocation differences resulted in maternal selection. Where natural selection can be described as the covariance between traits in an individual and fitness of that individual (Lande and Arnold 1983), maternal selection occurs when traits in the mother covary with offspring fitness (Kirkpatrick and Lande 1989). We therefore used multivariate selection analysis to derive maternal selection gradients that reflect the covariance between the amino acid profiles provided by mothers to the eggs and offspring performance traits that are related to offspring fitness. We found positive linear (directional) maternal selection operating on PC3 and PC4, suggesting allocation is positively associated with heavier hatchlings. Stabilizing maternal selection was also found to be acting on PC4 which suggests that this amino acid allocation profile should be favoured in relation to hatchling mass. We found positive directional maternal selection on PC3 and multivariate (disruptive) maternal selection acting on **m**_**1**,_ which is heavily loaded by PC3 with negative tradeoffs with PC1 and PC2. There was also directional maternal selection acting on PC4, as well as multivariate (stabilizing) maternal selection on **m**_**4**_, which is heavily loaded by PC4 and PC1 but has also positive loadings from PC2 and PC3. Overall, our results suggest selection on both acquisition and allocation, with perhaps greater selection on allocation.

The combination of disruptive selection acting on **m**_**1**_ and stabilizing selection acting on **m**_**4**_ gives the saddle-shaped fitness surface that translates into performance (fitness) peaks and troughs upon which hatchling mass from the different treatment groups can be plotted. When mean **m**_**1**_ and **m**_**4**_ scores from each treatment group are plotted on the performance surface, hatchlings from females from the Laboratory and Kentucky source populations feeding on milkweed were found to be closest to the peak of the performance surface, while hatchlings from females from the Laboratory and Kentucky source populations feeding on sunflower were further from the fitness performance peak. In other words, stabilizing selection acting on **m**_**4**_ suggests that the intermediate values for these amino acid allocation profiles are important in determining higher (better) performance parameters for hatchling mass in our two source populations of *O. fasciatus*. Allocation from milkweed is better optimized in both populations. Conversely, disruptive selection on **m**_**1**_ indicates that populations with lower mean values for **m**_**1**_ will demonstrate (relatively) poorer performance.

These results suggest that long-term selection for sunflower use, as experienced by the Laboratory population that has been maintained on sunflower for over 400 generations, has not resulted in more adaptive allocation patterns compared to the ancestral food of milkweed, even though individual performance on sunflower is greater in the Laboratory population (Newcombe et al. 2015). Consistent with the suggestion of Newcombe et al. (2015), individual responses evolved faster than the maternal effects associated with novel foods. This suggests that while maternal selection has influenced how females allocate amino acids to eggs, this maternal effect has not evolved equally in all populations. Moreover, none of the population means coincide directly with peak performance. It is possible that the evolution of this maternal effect may be constrained from reaching the phenotypic optima, although further work on the genetics of the amino acid composition of eggs in this species is needed to assess this possibility and to define the nature of the constraints.

While we have used multivariate selection analysis in exploring relationships between free amino acid profiles of eggs and offspring performance parameters, free amino acids are only one aspect of egg composition. Multivariate analyses are always limited by what is left out. Other components that females allocate to their eggs could supplement offspring development to confer maximum fitness. One major difference between milkweed and sunflower is the presence of cardenolides in the former but absent in the latter. We have shown that there is no difference between the source populations in allocation of cardenolides (Newcombe et al. 2013). Newly laid insect eggs also contain ecdysteroids of maternal origin (Sall et al. 1983). Insects synthesize ecdysteroids from plant sterols that are ingested during feeding. These sterols are usually dealkylated into cholesterol as a precursor for hormones. However, there is evidence that *O. fasciatus* is able to directly utilize sterols without having to dealkylate them first (Svoboda et al. 1977, 1983; Kelly et al. 1984). One plant sterol, campesterol, has been found to be a precursor for makisterone A in *O. fasciatus* and honey bees (Feldlaufer et al. 1985). Makisterone A is thought to be the predominant molting hormone in embryonated eggs of *O. fasciatus* (Kelly et al. 1984). Sunflower seeds have been shown to have around half the content of campesterol as milkweed seeds (Svoboda et al. 1983). It could be that a combination of insufficient campesterol and having the “wrong” amino acid profiles could impede developmental processes, resulting in smaller offspring. Further, other compounds such as proteins, lipids, glycogen (O'Brien et al. 2005), and carbohydrates (Bauerfeind and Fischer 2005) are essential to oogenesis and embryonic development. No single compound is likely to be the sole determinant of egg/offspring viability (Bauerfeind and Fischer 2005; Geister et al. 2008). Nevertheless, amino acids are essential and our work provides a starting point for examining their role as maternal effects and in adaptation.

How females allocate nutritional resources to egg production can give further insights into the relationships between nutritional ecology, resource allocation, and life-history traits (O'Brien et al. 2002; Boggs 2009) that influence insect population dynamics and host–plant interactions (Mousseau and Dingle 1991). In this study, we have shown how a multivariate approach to examining amino acid profiles of eggs may be one such method in determining how maternal nutritional experience can influence offspring life-history traits via maternal egg effects. Furthermore, it is possible to estimate the strength and direction of linear and nonlinear selective forces that may operate on a specific maternal trait (i.e., maternal allocation of amino acids) in determining how this may affect relative offspring fitness values. This highlights the potentially complex role of maternal effects in evolution and, given how maternal traits influence offspring fitness, why additional studies of maternal selection in other organisms are needed.

## Data Accessibility

Data deposited in the Dryad repository: doi:10.5061/dryad.68hp8.
